# Design and Validation of the Adaptation to Change Questionnaire: New Realities in Times of COVID-19

**DOI:** 10.3390/ijerph17155612

**Published:** 2020-08-04

**Authors:** María del Carmen Pérez-Fuentes, María del Mar Molero Jurado, África Martos Martínez, Elena Fernández-Martínez, Raquel Franco Valenzuela, Iván Herrera-Peco, Diana Jiménez-Rodríguez, Inmaculada Méndez Mateo, Azucena Santillán García, María del Mar Simón Márquez, José Jesús Gázquez Linares

**Affiliations:** 1 Department of Psychology, Faculty of Psychology, University of Almería, 04120 Almería, Spain; amm521@ual.es (Á.M.M.); msm112@ual.es (M.d.M.S.M.); 2 Department of Psychology, Faculty of Psychology, Universidad Politécnica y Artística del Paraguay, Asunción 1628, Paraguay; 3 SALBIS Research Group, Nursing and Physiotherapy Department, Faculty of Health Sciences, University of León, 24401 Ponferrada, Spain; elena.fernandez@unileon.es; 4 Hospital Universitario Mútua de Terrassa, 08001 Barcelona, Spain; rfranco@mutuaterrassa.cat; 5 Nursing Department, Health Sciences Collegue, Alfonso X El Sabio University, 28691 Madrid, Spain; iherrpec@uax.es; 6 Department of Nursing, Physiotherapy and Medicine, University of Almería, 04120 Almería, Spain; d.jimenez@ual.es; 7 Department of Evolutionary and Educational Psychology, University of Murcia, 30100 Murcia, Spain; inmamendez@um.es; 8 Department of Cardiology, Burgos University Hospital, 09006 Burgos, Spain; ebevidencia@gmail.com; 9 Department of Psychology, Universidad Autónoma de Chile, Providencia 7500000, Chile

**Keywords:** adaptability to change, COVID-19, general population, design, validation

## Abstract

Emotional and cognitive-behavioral factors influence people’s adaptability to change. Based on this premise, the objective of this study was to develop, evaluate and validate the Adaptation to Change Questionnaire (ADAPTA-10) for identifying those who show poor adaptability to adverse situations, such as those caused by COVID-19. This study was carried out in a sample of 1160 adults and produced a 10-item instrument with good reliability and validity indices. It is an effective tool useful in research and in clinical practice. Calculation tables are provided for the general Spanish population and by sex to evaluate adaptability to change. The two-dimensional structure proposed in the original model was confirmed. This instrument will enable the needs for adaptation to the new reality associated with COVID-19 to be detected and also other situations in which the subject becomes immersed which demand adaptation strategies in the new situation lived in.

## 1. Introduction

The disease caused by the coronavirus, SARS-CoV-2, called COVID-19 [1], was declared a pandemic by the World Health Organization in March 2020 [2] because of its rapid spread and high death toll [3].

The risk of this pandemic is not only a healthcare problem, but also has severe socioeconomic and psychological implications [4]. Therefore, the approach to the COVID-19 public healthcare emergency should try to minimize both the negative physical and psychological impacts of the virus [5]. Ignoring the immediate psychological effects of this global situation would have a disproportionate mid- to long-term impact [6]. Its severity and persistence are still unknown, and therefore, how long public restrictions and measures should be maintained is also unknown [7]. What is clear is that the transition to the new normality will be a process that will put the adaptability of every individual to the test.

### 1.1. Adaptation to Change

In situations of adversity, some individuals are at greater risk of developing psychological alterations, such as posttraumatic stress or biopsychosocial disorders, while others resist and adapt well [8]. In psychology, the concept of adaptation refers to functional change in response to environmental stimuli, whether in terms of sensorial, behavioral, cognitive or emotional functioning. These changes must provide benefits to the subject, improving adjustment to the current or future environment [9]. Positive adaptation to adversity is not a completely innate trait, so this ability can be learned and developed by actively reformulating life’s challenges [10]. From a perspective of virtue, based on committed action, the individual’s practical wisdom and courage, resilience and adaptability may be understood as the transformation of adversity into opportunity [11]. In research, psychological adaptation has often been analyzed in the scope of natural disasters due to the direct impact and high losses undergone by a high percentage of the population in such situations. These losses may be permanent or temporary, total or partial, depending on the capacity of strength of the individual and the number of stressors to cope with [12,13]. Women seem to adapt the worst to highly stressful events [14,15]. High financial losses and the death of loved ones are the two factors which have the strongest long-term repercussions on adaptation and subjective wellbeing of individuals [14,16]. Thus, psychological adaptability enables adjustment to or acceptance of difficult situations and is of great value in learning to struggle with the limitations of daily life [17]. On the contrary, absence of psychological adaptability has been linked to the presence of internalization (somatic complaints, anxiety or depression) and externalization (behavioral problems) symptoms [18]. Therefore, after a significant event, the wellbeing of an individual may not return to baseline or may take many years to do so, and the adaptation responses of each subject may differ enormously [19].

### 1.2. Factors in Adapting to Change

The hedonic adaptation model by Graham and Oswald [20] states that individuals tend to remain at a certain stable level of endogenous wellbeing, recovering from harmful events and becoming used to the good ones. Thus, when exogenous events threaten their wellbeing, people may recover, safeguarding their adjustment if they can control the situation to a certain extent through the flow of psychological resources for coping with it. Based on integration of individuals in their setting, this could generate different styles and strategies for approaching the situation [21]. According to the Threat Appraisal and Coping Theory [22], people who are exposed to stressful situations may respond with adaptive behavior which provides them with immediate and long-term wellbeing, or, with maladaptive coping, which distracts them or relieves them, making them feel good temporarily, but generating psychological distress later. According to Black and Hendy [23], the choice in many cases is related to the perceived ability to do something about the situation. Thus, although exposure to stressful factors may not always be avoidable, if one perceives that something can be done to change the situation, a more adaptive coping strategy, mainly related to proactive efforts to change the situation or its meaning, will be chosen [24,25]. Thus, staying in control of events goes beyond the resources for coping. The sense of control may be internal or external. Beliefs about stronger internal control buffer the effects of stressors, so people who feel more able to control stressful events adapt better, as they are more able to face them, even though they may be more strongly exposed to this type of event [22,26]. However, other authors note that although at first sight it might be thought that the internal locus of control is related directly with wellbeing, it is not always that way. When people face completely uncontrollable situations, maintaining a high perception of internal control of events could be a negative strategy for adaptation, generating emotional distress [27].

Another of the factors involved in an individual’s adaptability is tolerance to uncertainty. Uncertainty is present in daily life (e.g., Will it rain today?), at existential moments and important decisions (personal and professional), in relations with the world (e.g., the future and unemployment during an economic crisis) at significant times (e.g., illness of a family member) and in nature (e.g., fear of natural disasters in a prone geographic area). So, the way in which people perceive and cope with uncertainty is relevant to their adaptability [28]. Tolerance to uncertainty has been defined as the way in which people understand and process information in uncertain situations and how they respond with cognitive-behavioral and emotional reactions [29]. Repetitive, expected events do not usually awaken fear, but apprehension. Worry and uncertainty usually appear when the causes of an event cannot be explained [30]. Similarly, situations of psychological uncertainty are usually coupled with anxiety symptoms due to the agitation from anticipation of threat, and with stress, which refers to persistent irritability, impatience and tension; therefore, its management is important in order to lower psychological stress and ensure adjustment [31,32]. Along with stress management, the capacity for regulating emotions is fundamental to adapting later to indispensable situations [33,34]. Thus, people who show better ability to regulate their emotions have a stronger capacity for adapting and responding to a changing environment [35].

Depression is another emotional component to be kept in mind in an individual’s adaptation. Perception of the inability to cope with demands is linked to dysphoric feelings and depressive symptoms [36]. It has also been associated with cognitive inflexibility, which is transformed into problems for adapting reactions to new situations [37]. Thus, cognitive flexibility is another component to be considered in adaptation to change [38]. This refers to the ability to modify cognitive and behavioral strategies in response to changes in environmental demands [39]. Increase in psychological flexibility has been shown to diminish stress and anxiety that handicap effective response and provide benefits for wellbeing [40]. This capacity in turn depends on the ability to detect characteristics and changes in situations [38]. Monitoring conflicts is linked to the capacity for cognitive control, which facilitates assimilation and accommodation of conflict, and in turn, orientation toward specific objectives and resolution of potentially problematic or incongruent situations [41]. In a stressful situation, this could involve concentrating on information related to the threat and the one that leads to eliminating stressors, distancing oneself from nonessential information [38]. Thus, the mechanisms of control and conscious awareness enable detection and adaptation to situations in which information is conflictive [42].

The effects of awareness on the adaptability to change are mediated by perceived social support, which favors redefinition of stressful situations so they are not perceived as such or supply resources that enable the severity of such events to be reduced [43,44]. Thus, counting on strong perceived social support provides material sustenance and emotional comfort to people, in addition to helping them to reduce the negative evaluation of events, enabling them to alleviate distress and improve adaptation [45]. Along this line, the study by Koffer et al. [26] found that beliefs about control in stressful situations increase with age, postulating that this result could be due to the decrease in availability and efficacy of psychosocial resources.

**Figure 1:**
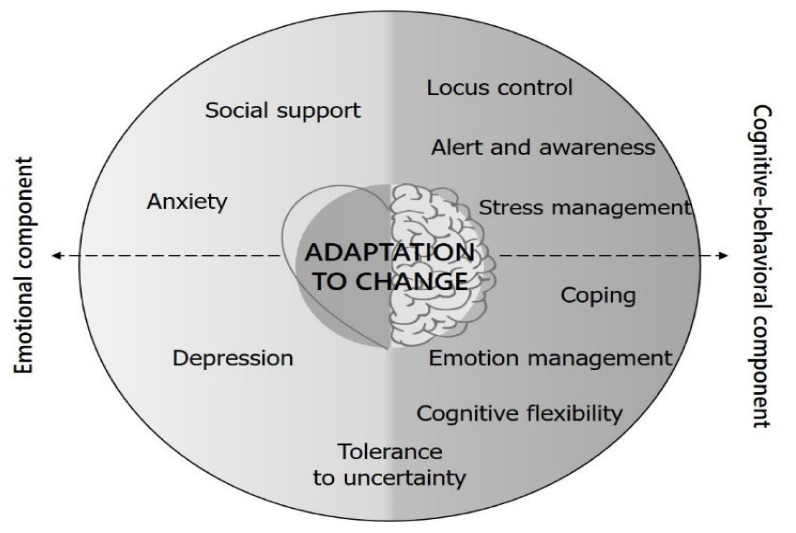
Theoretical explanatory model of the adaptability to change construct.

Interest in knowing the capacity of individuals to adapt to change has led to studies seeking to establish the cognitive and emotional dimensions giving rise to this variable. However, analysis of the factors that enable success in new situations and unexpected changes in one’s environment have focused mainly on the job context [46,47]. Hedonic adaptation to important life events has also been analyzed [48], but not to changes in environmental demands with less transcendence in life than the birth of a child or the death of a family member. Thus, to date, the factors determining adaptability to everyday events and demands have not been established. Therefore, the following model was hypothesized as a starting point for the design and validation of an evaluation scale for adaptability to change. The factors included on it are those mentioned above, differentiating between those that pertain to the emotional dimension because of their repercussion on feelings experienced during adaptation (social support, anxiety, stress and tolerance to uncertainty) and those pertaining to the cognitive-behavioral dimension because of its involvement in management, control and action on it (that is, stress management, locus of control, state of alertness, coping, emotional management, cognitive flexibility and tolerance to uncertainty) (Figure 1). The latter (tolerance to uncertainty) is included in both dimensions because it includes both emotional and cognitive responses [29].

### 1.3. Objective

There are many gaps in our knowledge of control, treatment or even socioeconomic effects derived from the COVID-19 pandemic [49]. Along with the strong perception of uncertainty and threat caused by the pandemic and by the new measures that must be adopted in daily life [50,51,52], they can affect behavioral efficacy and the capacity for management and coping [53]. Adaptability to change is fundamental to avoid psychological alterations linked to the accumulation of stressors [15], however, there is no instrument that specifically evaluates this capacity in the individual. Therefore, based on the model conceptualized above, and in view of its relevance for ensuring adjustment to change in daily scenarios, the objective of this study was to develop, evaluate and validate the Adaptation to Change Questionnaire.

## 2. Materials and Methods

### 2.1. Participants

The sample was made up of 1168 adult Spaniards. The questionnaire included control questions for detecting random or incongruent answers, which led to the elimination of eight subjects, so that the final sample was comprised of 1160 people. The mean age of the sample was 38.29 years (standard deviation (SD) = 13.71) in a range of 18 to 82. Of these, 69.9% (*n =* 811) were women and 30.1% (*n* = 349) were men, with mean ages of 37.05 (SD = 13.34) and 41.16 years (SD = 14.14), respectively.

### 2.2. Instruments

The sociodemographic data were collected in an ad hoc questionnaire, which included items on age, sex, marital status and education.

The General Health Questionnaire-28 (GHQ-28) [54,55] was used for evaluating general health and related functional symptoms. This questionnaire consists of 28 items grouped in four subscales with seven items each: Subscale A (somatic symptoms), Subscale B (anxiety and insomnia), Subscale C (social dysfunction) and Subscale D (severe depression). Each question has four gradually worsening answer choices. The subject must mark the answer chosen based on recent weeks.

The Adaptation to Change Questionnaire (ADAPTA-10) was designed to evaluate an individual’s adaptability to the demands of novel situations. This questionnaire is made up of 17 items related to the individual’s disposition to achieve successful adjustment to unknown situations or events. It includes items linked to emotions of distress and anxiety that could appear when faced with changes or others related to the capacity for controlling, managing and acting in different situations, that is: social support, anxiety experienced, depression, stress management, awareness and state of alertness, coping concentrated on the problem, tolerance to uncertainty, emotion management, mental flexibility and locus of control. The answers are rated on a five-point Likert-type scale (from “not at all” to “very much”).

### 2.3. Procedure

This cross-sectional study was done with snowball sampling carried out on social networks and instant messaging during the seventh and eighth week of confinement of the Spanish population, specifically from 1 to 12 May 2020. The participants filled out the tests individually in a time estimated at 5–10 min.

The stages that led to the conceptualization and development of the ADAPTA-10 Questionnaire for evaluating adaptability to change are described below. The study was approved by the University of Almeria Ethics Committee (UALBIO2020/032, 06-25-2020). All the subjects in the study participated voluntarily and gave their written informed consent prior to filling out the questionnaire, after being informed of the objectives of the research and the anonymous nature of their answers. The data were collected and processed respecting all of the rights and guarantees as provided for in EU Regulation 2016/679 and Organic Law 3/2018 of 5 December on Protection of Personal Information and guarantee of digital rights.

The questionnaire was implemented as a CAWI (Computer Aided Web Interviewing) interview, in which the participants expressly gave their consent by marking a box for the purpose before going to the questionnaire screen.

The first step was an analysis of the scientific literature on the subject of adaptability to change in an adult population. Search machines were used to collect studies that could contribute to the development of the items on the questionnaire.

After the review of the literature on the subject, experts were consulted to evaluate a first proposal of possible constructs for the final repertoire of indicators. The result of this stage was a list of constructs which we took as the starting point to develop the content of the items: social support, stress management, alertness, coping, tolerance to uncertainty, emotion management, locus of control, cognitive flexibility, anxiety and depression. Following this, a specific search was made on measurement of each of the proposals.

The next step was to write the items, which were in first person because it was to be a self-informed questionnaire. To check the intelligibility and clarity of this first set of items, a pilot questionnaire was drafted and distributed to a sample of 30 subjects selected by snowball sampling, all of them adults over 18 years of age. Then, the content and wording of the items were reviewed considering their observations, making minor modifications to reduce the answer bias or misunderstanding.

The questionnaire was comprised of 17 items and the answers for each item were rated on a five-point Likert-type scale (1 = not at all, 2 = a little, 3 = somewhat, 4 = quite a lot, 5 = very much).

Finally, the questionnaire was validated by administering it to a representative sample of adults (see sample characteristics in the section on Participants). Although the scale was designed with several theoretical constructs as the basis, we could not determine any latent factor models until the measurement structure was proven statistically based on the original theoretical model proposed.

### 2.4. Data Analysis

Data were analyzed in two stages following the validation steps recommended by Pérez-Fuentes et al. [56]. The first stage dealt with the study of the structure according to the original theoretical basis of the Adaptation to Change Scale. To approach this objective, the sample was first divided at random into two homogeneous independent subsamples. The first sample was used for calibration (*n* = 578) in the exploratory (EFA) and confirmatory factor analyses (CFA) of the proposed theoretical Adaptation to Change model. The confirmatory factor analysis was done for the original model taking the following indices of fit as measures: χ2/df (Degrees of freedom), Comparative Fit Index (CFI), Tucker-Lewis index (TLI) and Root Mean Square Error of Approximation (RMSEA), with their confidence interval (CI) at 90%. Values below five were considered acceptable for the χ2/df index [57] for the CFI and TLI over or near 0.90, and for the RMSEA, below or very near 0.08 [58]. As a general rule, fit of the model is considered to be good when the χ2/df ≤ 3, TLI > 0.90, CFI > 0.95 and RMSEA ≤ 0.05. The appropriate re-specifications were made of the model, which showed good indices of fit, considering theoretical and statistical criteria (change index, error of estimation, standardized error of measurement), but the model was not improved. The Akaike Information Criteria [59] was used for model selection. Then, the re-specified model was validated based on the second subsample (*n* = 583), used as the validation sample. The Cronbach’s Alpha [60], Spearman-Brown and intraclass correlation coefficient were used for the reliability analysis of the new scale.

Finally, in the second stage, an analysis was done that supports the invariance of the factor structure proposed across sex (men/women). First, the goodness of fit of these structures was tested in both subsamples separately (Model M0a—Men and Model M0b—Women). The result was four nested models which were evaluated: (A) Model 1: both samples together simultaneously with free estimation of the parameters, (B) Model 2: metric invariance shown, (C) Model 3: scalar invariance shown, (D) Model 4: strict invariance. With no criterion of consensus to determine the criteria to be used to evaluate the difference in fit between the nested models [61], for evaluation of fit, this study used the ΔCFI. Thus, the model is completely invariant if the ΔCFI is below 0.01 [62]. Similarly, the validity of the construct was evaluated by analyzing the correlation of the items and factors with other instruments that measure related aspects.

The analyses were performed using the e SPSS Statistical Package, version 23.0, for Windows (IBM, Armonk, NY, USA) and the AMOS 22 Program (IBM, Chicago, IL, USA).

## 3. Results

### 3.1. Preliminary Analyses 

In the first place, the data show that the items in the original ADAPTA-17GF (general factor) model have a distribution within the limits of normality according to the criteria of Finney and DiStefano [63], for whom 2 and 7 are the maximums permitted for skewness and kurtosis, which in our case were 1.28 and 2.74, respectively (Table 1).

### 3.2. Confirmatory Factor Analysis of the Original Model

Table 2 shows the fit of the various questionnaire models according to the original ADAPTA 17 model (with a general adaptation factor and two other factors: emotional and cognitive-behavioral). This model was re-specified considering theoretical and statistical criteria (indices of change, errors of estimation, standardized errors of measurement).

It may be observed that both the original 17-item model and the 12-item model show values that could be improved. The two-factor model with a general adaptation factor and 10 items is the best one after analysis. Thus, the ADAPTA-10GF Model showed much better fit in the calibration sample. There is also a smaller difference between the AIC default model = 141,996 and the AIC Saturated model = 110,000, showing that it is probably the best model according to the Akaike model selection criteria.

### 3.3. Exploratory Factor Analysis of the ADAPTA-10GF Model

**Figure 2:**
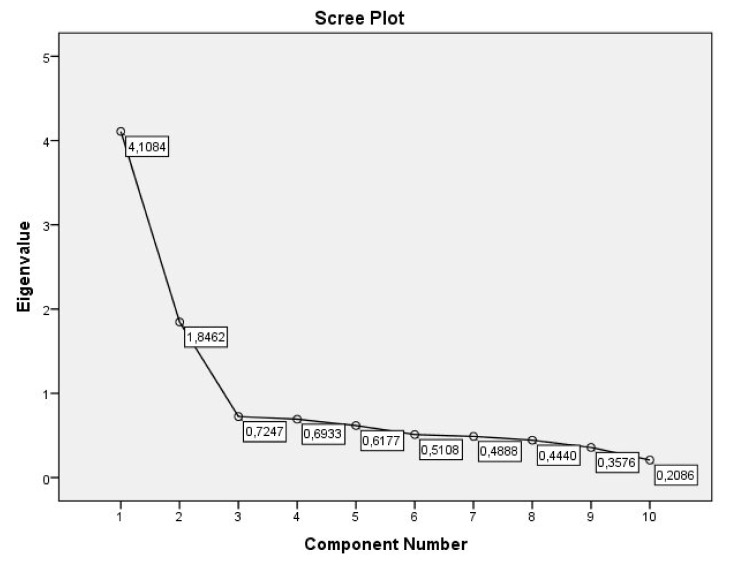
Scree plot of factor analysis for the ADAPTA-10 GF Model.

The Principal Components Analysis revealed the existence of two components with eigenvalues over 1. The Scree Test showed the presence of two factors (Figure 2). Thus, we see in that in Table 3, there are two components corresponding to the Emotional Component and the Cognitive-Behavioral Component in the original model, with five items each, all with weights over 0.65, and explaining 59.55% of the variance (Table 3).

Reliability of the model was analyzed with the Spearman-Brown coefficient *p* = 0.73 and the Cronbach’s Alpha, which for the whole scale was α = 0.84. The intraclass correlation coefficient (ICC) and its confidence interval (CI) were used for the analysis of temporal stability, with the following results for adaptation to change: 0.84 (CI = 0.82–0.86).

**Figure 3:**
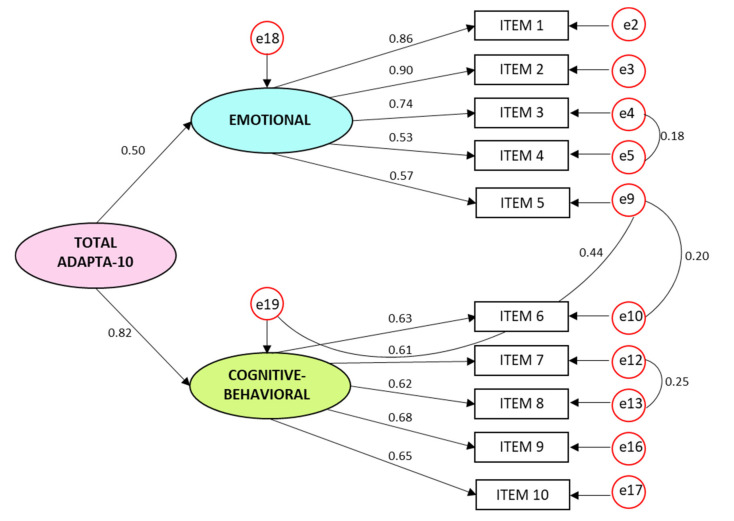
Confirmatory factor analysis ADAPTA-10GF Model (*N* = 583).

Confirmatory Factor Analysis data for the model proposed (Figure 3) with the validation sample (*n* = 583) showed the following measures of fit: *χ^2^/df =* 3.21, CFI = 0.970, TLI = 0.956 and RMSEA= 0.062 (0.048–0.076), which were all adequate.

The values in Table 4 for the six different models in the analysis of variance across sex show that in all cases, the ΔCFI is less than 0.01, and therefore, configural, metric, strict and strong invariance are accepted.

**Figure 4:**
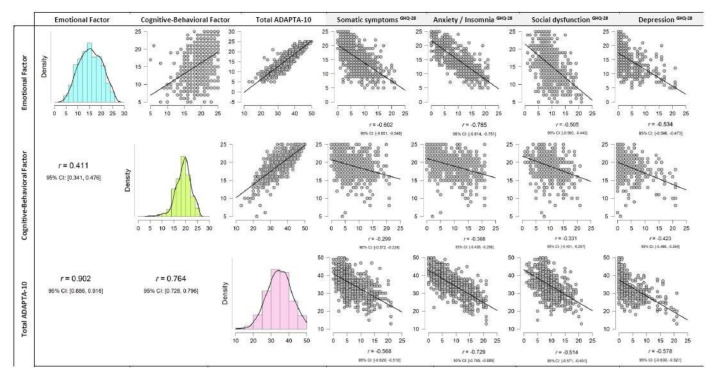
ADAPTA-10 questionnaire correlations and General Health Questionnaire-28.

With regard to construct validity, Figure 4 shows that the correlations in the direct scores on the GHQ-28 health questionnaires and the ADAPTA-10 questionnaire are significant (*p* < 0.01) and negative in all cases, backing the validity of the ADAPTA-10 construct. A higher score on the GHQ-28 shows more problems in each of the health dimensions.

## 4. Discussion

Adaptation to change is an important concept in psychology, as it depends on the adjustment of functioning and the responses with which one copes with the diversity of environmental demands [9,17]. Its absence has been related to psychological alterations [18]. Given the speed with which daily scenarios vary and the number of novel demands which must be coped with in short periods of time, knowing the effects of the capacity of adaptation to change of the population may be beneficial to both immediate and long-term psychological health [6]. In this respect, models have been proposed to establish the dimensions and factors that intervene in the process of adaptation to change, but linked to transcendental life events (such as the appearance of a disability, birth of a child or death of a spouse) or employment demands [46,47]. This study proposed construction of a model of adaptation to change in everyday events and circumstances, which would enable a scale to be designed for evaluating the response to challenges and changing circumstances.

In the validation of the Adaptation to Change Questionnaire, ADAPTA-10, in the general population, the exploratory and confirmatory factor analyses showed the existence of the two dimensions previously found in the original model: emotional (linked to feelings that arise during adaptation) and cognitive-behavioral (related to cognitive management and behaviors for that purpose). Based on the factors analyzed and according to Bjorklund [9], it seems that the capacity for adapting to change includes both types of response, which would be in line with the model that showed the best fit. However, although the index of this two-factor model was adequate, after performing the corresponding re-specifications following theoretical and statistical criteria, seven items were eliminated from different factors in the original model. Specifically, items pertaining to the social support, stress management, locus of control and flexibility items were eliminated. In social support, the item eliminated may have been related, as mentioned by Kim et al. [43], with support being a mediator in the individual adaptation process, positively promoting one’s resources to cope with challenges, but not as a factor directly involved in this capacity. With regard to the stress management item, it may not be part of the validation process, since in situations in which the response must be rapidly modified or adjusted, a certain level of stress can eliminate lethargy or paralysis and generate the drive necessary to make the appropriate modifications. Furthermore, as items referring to emotional management were entered, negative thoughts and feelings that could arise with the appearance of stress (such as anxiety, irritability and so forth) and diminish the capacity for adaptation, could have been covered by that factor. Concerning the locus of control, even though items related to internal and external control which could diminish the capacity for adaptation were included, they did not form part of the final model. This may have been due to the perception of control and cause of events, although generating a stronger feeling of capacity for managing situations [22,26] may not be directly related to one’s possibility to adapt. That is, the capacity for adjusting to daily situations may be independent of the control that one feels one has over them. Finally, the item referring to cognitive flexibility was also eliminated from the two-factor, ten-item model. This result may be due to its being a relevant factor or variable with a heavy weight which acts as a mediator in the adaptation process but does not affect it directly. However, due to the wide presence in the scientific literature of this factor as the one which provides the most possibility of modifying strategies and reactions to meet changing demands to ensure adjustment [37,38,39,40], this relationship must be reexamined in the future.

Therefore, based on the results, two dimensions were extracted from the ADAPTA-10 questionnaire. The first of these, the emotional dimension, would be linked to feelings experienced during adaptation. The five items that form part of this dimension pertain to the anxiety, depression and tolerance to uncertainty factors. Studies have shown the presence of anxiety symptoms, such as agitation from the need for adaptation to new demands [30]. Feelings of dysphoria and depressive symptoms are also present when the challenges one is faced with put the capacity to cope effectively with them to the test [36]. The cognitive-behavioral dimension refers to competence for managing and undertaking action to respond appropriately to daily situations that can be challenging. State of alertness enables conflictive situations to be detected and directs one’s attention toward specific objectives that must be met or problems that must be solved [41]. Coping concentrated on the problem means that efforts made are directly related to modifying the situation or its meaning, enabling its functional and adaptive management [24,25]. The emotional management factor may facilitate the regulation of negative feelings that appear because of the uncertainty, threat or perceived inability [35]. In the end, tolerance to uncertainty, as mentioned, formed part of the two dimensions through two different items. These referred to the emotional reaction and behavior in situations where one does not have all the information [29].

Thus, the Adaptation to Change Questionnaire, ADAPTA-10, is a short instrument, easily applied, which enables finding out the individual’s ability to adjust the best way possible to new demands based on two dimensions. Even so, there are some limitations. It should be mentioned that most of the sample was made up of women, although the questionnaire showed good invariance across sex, and could be reflecting populational characteristics in Spain. Another limitation derived from the way data were collected, as the mean age was low with respect to the reality of the Spanish population, since fewer older people use the new technology tools with which the questionnaire was publicized and data were collected. In future, when the health situation so permits, these age groups should be approached to include more such subjects, although as observed in the section on participants, older people also answered correctly. Future research could validate our findings even more through the use of a more general sample. Another of the limitations is derived from the study design, because, as a cross-sectional study, there were variables which could not be controlled. The performance of a longitudinal study would solve this limitation by evaluating longitudinal invariance of the questionnaire.

Although this is not a tool specific to COVID-19, it is a contextualized tool, so it would be necessary to analyze it again when the special situation of the health emergency ends. Meanwhile, its use along with other instruments evaluating psychological variables in the context of the COVID-19 pandemic can have very useful clinical applications. Evaluation of threat [52] or perceived risk from COVID-19 [64,65,66], combined with the capacity for adaptation to change, can help develop risk profiles and mental health protection measures in the mid- to long-term.

## 5. Conclusions

The Adaptation to Change Questionnaire, ADAPTA-10, for the general population possesses favorable psychometric properties. The internal consistency of both the total scale and the two factors (emotional and cognitive-behavioral) is adequate, and therefore, the general fit is acceptable. However, it is recommended that the goodness and fit of the model for testing the psychometric properties of the instrument continue to be analyzed in other specific collectives or contexts. The construction of this scale can contribute to the analysis of the consequences associated with the presence of low adaptability to change. The analysis of this construct emerged during the pandemic from the SARS-CoV-2 coronavirus, which has been mentioned by various authors as both a physical and psychological health emergency due to the high impact of the illness on people’s daily lives. This is because, to a greater or lesser extent, everyone must adapt to a highly changing environment. The absence of the capacity to recover one’s previous state of wellbeing in transcendental life circumstances has shown to have long-term psychological effects. This scale can provide further knowledge of this ability and its repercussions in uncertain everyday situations, not necessarily linked to such events. It can also be valid for establishing the level of this variable in individuals, enabling development of intervention programs to strengthen adaptability, and thereby, promote better adjustment to demands. Therefore, the psychometric indicators, both for the factors and the global scale, reveal that it is a reliable, valid measurement instrument for use in research. Likewise, it is thought that it can be of maximum usefulness for the prevention and early diagnosis of problems related to mental health (such as depression, anxiety, development of health-risk behaviors or use of maladaptive coping strategies) in the general population derived from poor adaptation to adverse situations, similar to those triggered by the COVID-19 pandemic.

## Figures and Tables

**Table 1:** Descriptive statistics. Calibration sample (*n* = 578). SD: standard deviation, Std. Error: standard error.

Items	*n*	M	SD	Skewness		Kurtosis	
				Statistics	Std. Error	Statistic	Std. Error
ADAPTA 1	578	4.3	0.83	−1.21	0.10	1.41	0.20
ADAPTA 2	578	3.00	1.19	0.03	0.10	−0.85	0.20
ADAPTA 3	578	2.95	1.20	0.05	0.10	−0.87	0.20
ADAPTA 4	578	3.22	1.26	−0.22	0.10	−0.95	0.20
ADAPTA 5	578	3.57	1.20	−0.44	0.10	−0.78	0.20
ADAPTA 6	578	4.00	1.01	−0.90	0.10	0.24	0.20
ADAPTA 7	578	2.73	1.28	0.24	0.10	−0.94	0.20
ADAPTA 8	578	3.75	1.07	−0.79	0.10	0.17	0.20
ADAPTA 9	578	3.20	1.18	−0.22	0.10	−0.80	0.20
ADAPTA 10	578	3.59	0.94	−0.41	0.10	−0.12	0.20
ADAPTA 11	578	3.66	1.02	−0.64	0.10	0.00	0.20
ADAPTA 12	578	4.30	0.73	−1.16	0.10	2.34	0.20
ADAPTA 13	578	4.30	0.75	−1.28	0.10	2.74	0.20
ADAPTA 14	578	3.65	1.04	−0.79	0.10	0.19	0.20
ADAPTA 15	578	4.06	0.93	−1.09	0.10	1.07	0.20
ADAPTA 16	578	4.01	0.83	−0.83	0.10	0.95	0.20
ADAPTA 17	578	3.54	0.94	−0.44	0.10	−0.09	0.20

**Table 2:** Fit indices for the models proposed (calibration sample *n* = 578).

Model			CFI	TLI	RMSEA		
	χ^2^ (df)	χ^2^/df			RMSEA	CI90%	
					Lower	Upper	
Original ADAPTA-17GF Model	690,331 (118)	5.85	0.801	0.770	0.092	0.085	0.098
ADAPTA-12GF Model	432,200 (53)	8.154	0.846	0.808	0.111	0.102	0.126
ADAPTA-10GF Model	91,996 (30)	3.066	0.969	0.954	0.06	0.046	0.074
ADAPTA-10 Model	106,175 (31)	3.425	0.963	0.946	0.065	0.052	0.079

CFI = Comparative fit index; TLI = Tucker-Lewis index; RMR = Root mean square residual; RMSEA = Root Mean Square Error of Approximation; CI = Confidence Interval; *df* = Degrees of freedom; Est. = Estimation.

**Table 3:** Factor structure, communalities (*h^2^*) eigenvalues, Cronbach’s alpha and percentage of explained variance (*n* = 583). Extraction method: Factoring of principal components.

Items	F1	F2	*h^2^*
Item 1. I feel nervous, tense and irritable	0.867		0.752
Item 2. I am worried and it’s hard for me to relax	0.887		0.794
Item 3. I feel like I don’t have enough energy to cope with everyday life	0.824		0.678
Item 4. I’ve lost hope of recovering my normal life	0.676		0.458
Item 5. I’m calm, but I don’t know what is going to happen at any moment	0.671	0.432	0.498
Item 6. I can act in any situation, even though I don’t have all the information		0.695	0.509
Item 7. I consider myself smart, I am aware of what is happening around me		0.774	0.606
Item 8. When I have a problem, I make an effort to solve it		0.773	0.604
Item 9. I recognize my emotions, those of others and act accordingly		0.756	0.575
Item 10. I control my emotions when I think they could make things worse for me	0.446	0.652	0.482
Percentage of explained variance	41.08%	18.46%	
Kaiser-Meyer-Olkin		0.85	
Barlett’s sphericity	χ^2^(45) = 2276.17, *p* < 0.000		
Cronbach’s Alpha	0.85	0.78	0.84

**Table 4:** Multigroup analysis of invariance across sex (men/women).

Model	χ^2^	df	χ^2^ / df	Δχ^2^	CFI	ΔCFI	IFI	RMSEA (IC 90%)
M0a (men)	124.169 (*p* = 0.000)	60	2.069		0.971		0.971	0.043 (0.032–0.054)
M0b (women)	124.169 (*p* = 0.000)	60	2.069		0.971		0.971	0.043 (0.032–0.054)
M1 (base model)	138.387 (*p* = 0.000)	68	2.035	0.034	0.968	-	0.968	0.042 (0.032–0.052)
M2 (FS)	139.905 (*p* = 0.000)	69	2.027	0.008	0.968	-	0.968	0. 042 (0.032–0.052)
M3 (FS + Int)	141.494 (*p* = 0.000)	71	1.993	0.034	0.968	-	0.968	0. 042 (0.032–0.052)
M4 (FS + Int + Err)	156.330 (*p* = 0.000)	85	1.839	0.154	0.968	-	0.968	0. 041 (0.031–0.051)

FS = Factor saturations, Int = Intercepts, Err = Errors.
